# Scallop mantle toxin induces apoptosis in liver tissues of mice

**DOI:** 10.1002/fsn3.1608

**Published:** 2020-05-20

**Authors:** Takahide Kariya, Yasushi Hasegawa

**Affiliations:** ^1^ College of Environmental Technology Muroran Institute of Technology Muroran Japan

**Keywords:** food, liver, scallop mantle, toxin

## Abstract

We had previously shown that the intake of scallop mantle tissue resulted in the death of mice and rats. In this study, we investigated the liver injury caused by mantle tissue to clarify the mechanism behind its toxicity. Mantle toxin increased lipid peroxidation and decreased the reductive thiol content as well as the DPPH radical scavenging activity, catalase activity, and glutathione content in the liver of the mice. These results suggested that the mantle tissue diet caused oxidative stress through the decrease in antioxidants. In addition, mantle toxin increased the mRNA expression of endoplasmic reticulum (ER) stress‐ and inflammation‐induced genes and the protein expression of caspase‐3 and Bax (which induce apoptosis), suggesting that the mantle tissue diet causes apoptosis through oxidative stress, ER stress, and inflammation in the liver tissue. Such liver injury may be an essential cause of the rodent demise.

## Graphical abstract

We had previously shown that the intake of scallop mantle tissue resulted in the death of mice and rats. In this study, we have investigated how liver tissue injuries are caused by the mantle toxin. Our results showed that the mantle toxin caused liver tissue apoptosis through the induction of oxidative stress, ER stress, and inflammation in the organ.

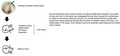

## 1 INTRODUCTION

Scallops, a major fishery product in Japan, feed on toxic marine algae such as dinoflagellates and thus accumulate marine toxins in their digestive glands. Although marine shellfish with toxins that cause diarrhetic shellfish poisoning (DSP), paralytic shellfish poisoning (PSP), neurotoxic shellfish poisoning, and amnesic shellfish poisoning have been reported (Farabegoli, Blanco, Rodríguez, Vieites, & Cabado, 2018; Smith & Swoboda, 2018), only two types of PSP‐ and DSP‐causing toxins have been detected in Japan. Toxins that cause PSP inhibit sodium ion influx by binding to the voltage‐gated sodium channel (Mattei & Legros, 2014) and inhibit generation of the action potential in neurons and muscle cells. Although DSP‐causing toxins are powerful inhibitors of phosphatases, which play important roles in signal transduction in the cells, it remains unclear whether that inhibitory activity is involved directly in their toxicity (Munday, 2013). We had previously reported that the intake of scallop mantle tissue caused the death of mice and rats without paralysis and diarrhea, where the toxin involved was different from DSP‐ and PSP‐causing ones (Hasegawa, Itagaki, Konno, & Hasegawa, 2018).

The liver is the main organ that transforms and clears chemicals and toxicants, making it susceptible to the toxicity of many agents. For example, galactosamine causes liver injury through its induction of inflammation and oxidative stress (Das, Ghosh, Roy, & Sil, 2012). Carbon tetrachloride (CCl_4_) damages the liver through its mediation of lipid peroxidation and subsequent inflammation (Fu, Zheng, Lin, Ryerse, & Chen, 2008). Treatment with both *N*,*N*‐dimethylformamide and CCl_4_ injures hepatocytes by increasing endoplasmic reticulum (ER) stress (Kim et al., 2010). Excessive amounts of iron also cause oxidative stress and liver injury, although an optimum level of iron is always maintained in the cells (Zang et al., 2012). Chronic ethanol exposure is known to cause liver injury, with increased ER stress, oxidative stress, and inflammation (Ambade & Mandrekar, 2012). Thus, many hepatotoxicants cause liver injury through their induction of oxidative stress, inflammation, ER stress, and apoptosis.

There are few studies on the toxic effects of PSP‐ and DSP‐causing toxins in the liver after oral administration. One study found that although oral treatment with yessotoxin (a toxin that causes DSP) did not induce any significant difference in the expression of the liver enzymes aspartate aminotransferase (AST) and alanine aminotransferase (ALT), which typically increase in hepatic injury, the oral administration of okadaic acid (another DSP‐causing toxin) caused a statistically significant dose‐dependent increase in the ALT and AST levels (Tubaro et al., 2004). Chronic ingestion of saxitoxin (one of the PSP‐causing toxins) decreased the expression of the antioxidants, resulting in oxidative stress in the liver (Ramos, Diehla, Santos, Monserrat, & Yunes, 2014). We showed previously that distinct cell shrinkage was observed in the liver tissue of the rats fed a diet containing mantle epithelial cell layer by histology (Hasegawa et al., 2018). In this study, we investigated several indicators of liver injury in mice fed a diet of toxic mantle tissue in order to clarify the mechanisms by which the toxin causes death of the animals.

## 2 MATERIALS AND METHODS

### 2.1 Materials

Scallops (*Patinopecten yessoensis*) were purchased at a market in Mutsu Bay for preparation of the mantle tissue. Antibodies against beta (β)‐actin, procaspase 3, caspase 3, B‐cell lymphoma 2 (Bcl‐2), Bcl‐2‐like protein (Bax), c‐Jun N‐terminal kinase (JNK), T183‐phosphorylated JNK (p‐JNK), glycogen synthase kinase‐3 beta (GSK‐3β), and S9‐phosphorylated GSK‐3β (p‐GSK‐3β) were purchased from Biorbyt.

### 2.2 Experimental animals and diets

Male ICR mice (4 weeks old) were purchased from CLEA (Tokyo, Japan) and acclimated on a normal diet for a week. The mice were then divided randomly into two groups (5 or 6 mice per group) and fed a diet without (control diet) or with 3% mantle tissue including epithelial cell layer (mantle diet) for 4–8 weeks. The contents of each diet are shown in Table 1. The mice were fed 4 g of each diet every day, and the amount of food consumption was measured daily as an index of toxicity (Hasegawa et al., 2018). Blood was collected from the tail vein every week, and the glucose concentration was measured using a glucometer and test strips (Abbott). At 6 weeks, the mice were anesthetized and the liver was quickly excised and stored at −80°C until use. The animal experiments (approval number H29KS04) were carried out according to the Experimental Animal Care Guidelines of the Muroran Institute of Technology.

**Table 1:** Primer sequences used in semiquantitative RT‐PCR

Gene	Forward primer	Reverse primer
β‐actin	5′‐GGCTGTATTCCCCTCCATCG−3′	5′‐CCAGTTGGTAACAATGCCATGT−3′
CuZn‐SOD	5′‐CGGATGAAGAGAGGCATGTT−3′	5′‐CACCTTTGCCCAAGTCATCT−3′
Mn‐SOD	5′‐GCACATTAACGCGCAGATCA−3′	5′‐AGCCTCCAGCAACTCTCCTT−3′
HO−1	5′‐TTCAGAAGGGTCAGGTGTCC−3′	5′‐CAGTGAGGCCCATACCAGAA−3′
ATF4	5′‐GAGCTTCCTGAACAGCGAAGT−3′	5′‐TGGCCACCTCCAGATAGTCAT−3′
GRP78	5′‐GGAAAGAAGGTTACCCATGC−3′	5′‐GGAACAGGTCCATGTTCAGC−3′
CHOP	5′‐TCACTACTCTTGACCCTGCG−3′	5′‐ACTGACCACTCTGTTTCCGT−3′
G6Pase	5′‐GACCTCCTGTGGACTTTGGA−3′	5′‐AGTTCTCCCTTGCAGCTCTT−3′
PEPCK	5′‐AGAACAAGGAGTGGAGACCG	5′‐TCCTACAAACACCCCATGCT−3′
SOCS3	5′‐CACCTACTGAACCCTCCTCC−3′	5′‐AGAGATGCTGAAGAGTGGCC−3′
TNF	5′‐TACTGAACTTCGGGGTGATTGGTCC−3′	5′‐CAGCCTTGTCCCTTGAAGAGAACC−3′
IL−1	5′‐ACTCATTGTGGCTGTGGAGA−3′	5′‐TTGTTCATCTCGGAGCCTGT−3′

### 2.3 Semiquantitative reverse transcription polymerase chain reaction analysis

Total RNA from the liver tissue was prepared using the RNAiso Plus reagent (Takara) and amplified with the semiquantitative reverse transcription polymerase chain reaction (RT‐PCR). The primer sequences used are shown in Table 2. After agarose gel electrophoresis, the quantity of the amplified PCR products was estimated using ImageJ software and presented as a ratio to the β‐actin content. The PCR cycles were selected on the basis of the relationship between the number of amplification cycles and the number of PCR products.

**Table 2:** Composition of control diet and mantle diet

	Control diet (%)	Mantle diet (%)
Casein	21	20
Corn starch	16.5	15
Cellulose	5.5	5
Mineral mixture	3.5	3.5
Vitamins mixture	1	1
Free base L‐cysteine	0.3	0.3
Choline bitartrate	0.2	0.2
Soybean oil	5	5
Mantle tissue	0	3
Sucrose	50	50
Total	103	103

### 2.4 Western blot analysis

Western blotting was performed as described previously (Hasegawa et al., 2018). In brief, the liver tissue was first homogenized in deionized water, and sodium dodecyl sulfate (SDS) was then added to a concentration of 2%. After the addition of bromophenol, SDS polyacrylamide gel electrophoresis (Laemmli, 1970) of the mixture was carried out, following which the protein bands were electrotransferred onto a polyvinylidene difluoride membrane. The membrane was incubated with 5% skim milk (w/v) in a solution containing 0.5 M NaCl, 20 mM Tris‐HCl (pH 7.5), and 0.05% Tween 20 (solution A), for 2–6 hr at room temperature, and then reacted overnight with antibodies against β‐actin, procaspase 3, caspase 3, Bcl‐2, Bax, JNK, p‐JNK, GSK‐3β, or p‐GSK‐3β. Thereafter, the membrane was treated with an alkaline phosphatase‐conjugated secondary antibody for 2 hr and then incubated with 5‐bromo‐4‐chloro‐3‐indolyl phosphate and nitroblue tetrazolium for color development. The band intensities were estimated using ImageJ software.

### 2.5 Antioxidative activities

After the liver tissue had been homogenized in a solution containing 20% sucrose, the supernatant was used as a liver extract for determining its antioxidative activities. For the 2,2‐diphenyl‐1‐picrylhydrazy (DPPH) radical scavenging assay, a solution of 0.8 mg/ml DPPH in 50% ethanol was mixed with the liver extract and the decrease in absorbance at 517 nm was then measured for 30 min (Kano, Takayanagi, Harada, Makino, & Ishikawa, 2005).

The content of sulfhydryl (SH) groups was determined according to the procedure of Ou, Kwork, Wang, and Bao (2004). In brief, 2 ml of 10 mM 5,5ʹ‐dithiobis(2‐nitrobenzoic acid), 100 ml of 10 mM ethylenediaminetetraacetic acid (EDTA), and 5 ml of the liver extract were mixed and incubated at room temperature. After 15 min, the absorbance at 412 nm was measured.

The malondialdehyde (MDA) content was determined as an index of lipid peroxidation. In brief, the liver extract was mixed with 50% trichloroacetic acid and the mixture was then centrifuged at 14,000 × *g* for 10 min. The supernatant was incubated with 0.67% thiobarbituric acid at 100°C for 10 min, and the absorbance at 540 nm was then measured (Shah, Kumar, Verma, & Dubey, 2001).

The glutathione content was measured with a BIOXY TECH GSH‐400 kit (OXIS International) according to the manufacturer's protocol. The glutathione peroxidase activity was determined in an assay mixture that contained 20 μl of 0.5 M potassium phosphate (pH 7.0), 100 μl of liver extract, 200 μl of 2 mM NADPH, and 200 μl of deionized water. After incubation at 37°C for 5 min, 100 μl of 15 mM cumen hydroperoxide was added and the change in absorbance at 340 nm was monitored (Klivenyi et al., 2000).

The glutathione reductase activity in the liver extract was measured according to the method described by Mannervik (1999). The enzyme assay mixture contained 500 μl of 0.2 M potassium phosphate (pH 7.0), 100 μl of liver extract, 100 μl of 0.2 M KCl, 100 μl of 10 mM EDTA, and 100 μl of deionized water. Upon the addition of 50 μl of 20 mM glutathione disulfide and 50 μl of 2 mM NADH, the change in absorbance at 340 nm was monitored.

The glutathione *S*‐transferase activity was determined in an assay mixture that contained 500 μl of 0.2 M potassium phosphate (pH 7.0), 100 μl of 10 mM glutathione, and 100 μl of 10 mM 1‐chloro‐2,4‐dinitrobenzene. The reaction was initiated by the addition of 100 μl of liver extract, and the absorbance at 340 nm was measured (Vontas, Enayati, Small, & Hemingway, 2000).

The catalase activity was measured in a solution containing 50 μl of 1 M Tris‐HCl (pH 8.0), 50 μl of 5 mM EDTA, 900 μl of 10 mM H_2_O_2_, and 30 μl of deionized water, according to the method of Hadwan and Abed (2016). Upon the addition of 20 μl of liver extract, the decrease in the absorbance of H_2_O_2_ at 240 nm was measured.

### 2.6 Other activities

The glycogen content was measured as described previously (Hasegawa et al., 2018). After extraction of the liver tissue with 10% trichloroacetic acid, ethanol was added to a concentration of 80%. The precipitate was collected and dissolved in deionized water, and the solution was then mixed with anthrone reagent. After incubation at 100°C for 10 min, the absorbance at 620 nm was measured.

AST and ALT were measured using AST and ALT assay kit (Fuji film).

### 2.7 Statistical analysis

Data are expressed as the mean ± standard deviation (*SD*) for each group, and statistical significance was analyzed using Student's *t* test.

## 3 RESULTS

### 3.1 Estimation of oxidative stress in the liver

**Figure 1:**
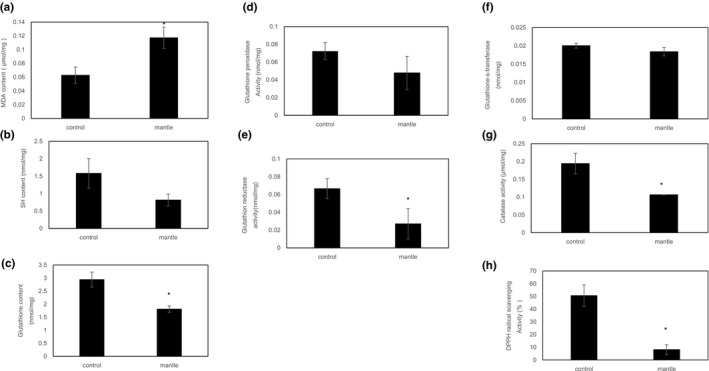
Effect of mantle tissue ingestion on oxidative injury and antioxidative activity in liver tissue. After the liver tissue of mice fed a diet containing mantle tissue or that of mice fed a control diet was extracted with 20% sucrose solution, the MDA (a) and reactive thiol (b) contents were measured. The glutathione content (c), glutathione peroxidase activity (d), glutathione reductase activity (e), glutathione transferase activity (f), catalase activity (g), and DPPH radical scavenging activity (h) were measured as described in the Materials and Methods section. Bars represent the *SD*. *p < .05 relative to the control

**Figure 2:**
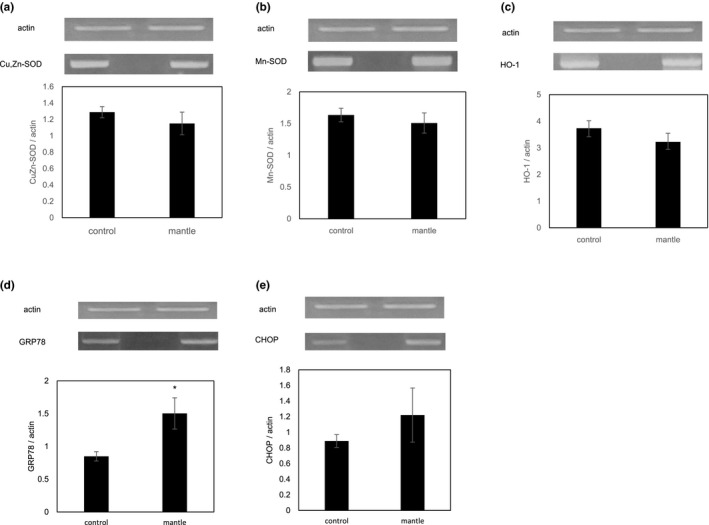
Effect of mantle tissue ingestion on the mRNA expression of antioxidants and ER stress‐induced genes. Liver tissue was excised from mice fed a diet containing mantle tissue and those fed a control diet, respectively. The mRNA expression levels of antioxidants (a–c) and ER stress‐induced genes (d, e) were estimated by semiquantitative RT‐PCR. (a) Cu/Zn‐SOD, (b) Mn‐SOD, (c) *HO‐1*, (d) *GRP78*, and (e) *CHOP*. Bars represent the *SD*. *p < .05 relative to the control

We showed previously that diet containing 0.2% mantle epithelial cell layer causes the death of rats (Hasegawa et al., 2018). In this study, we used mantle tissue including epithelial cell layer because a diet containing 3% mantle tissue showed the same toxicity as a diet containing 0.2% mantle epithelial cell layer (data not shown). Mice fed the mantle diet had significantly higher serum activities of AST and ALT activities compared to the control (236 IU/L versus 89 IU/L for AST and 105 IU/L versus 27 IU/L for ALT) as described previously (Hasegawa et al., 2018). To investigate whether oxidative stress occurred in the liver, the MDA content (indicative of the extent of lipid peroxidation) and SH content (indicative of the oxidative damage to proteins) were measured. Lipid peroxidation was significantly increased and the SH content decreased in the liver tissue of the mice fed the mantle diet (Figure 1). To confirm this result, we measured the activities of several antioxidants in the liver. The glutathione content, DPPH radical scavenging, catalase, and glutathione reductase activities were all decreased, and the glutathione peroxidase and glutathione transferase activities showed a decreasing tendency in the liver of the mice fed the mantle diet. Semiquantitative RT‐PCR analysis also showed that the mRNA expression levels of Cu,Zn superoxide dismutase (*SOD1*), Mn‐dependent SOD (*SOD2*), and heme oxygenase‐1 (*HO‐1*) had a tendency to decrease slightly although significant differences were not found (Figure 2). These results show that the mantle diet causes oxidative stress by decreasing the antioxidants in the liver tissue.

### 3.2 Endoplasmic reticulum stress and inflammation in the liver

**Figure 3:**
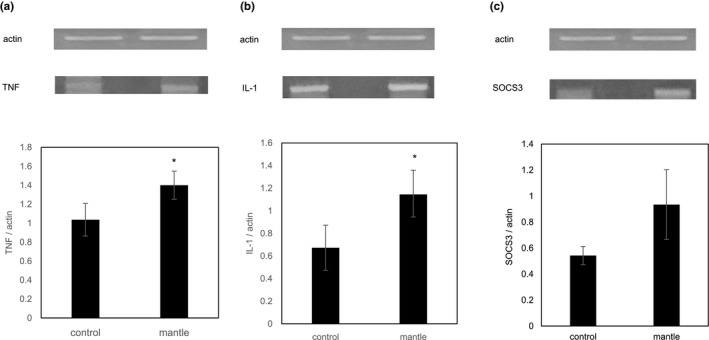
Effect of mantle tissue ingestion on the mRNA expression of inflammation‐induced genes in the liver. Liver tissue was excised from mice fed a diet containing mantle tissue and those fed a control diet, respectively. The mRNA expression levels of inflammation‐induced genes (a–c) were investigated by semiquantitative RT‐PCR. (a) *TNF‐α*, (b) *IL‐1α*, and (c) *SOCS3*. Bars represent the *SD*. *p < .05 relative to the control

Because ER stress is reportedly involved in the pathogenesis of liver injury (Malhi & Kaufman, 2011), we measured the mRNA expression levels of ER stress‐induced genes in the liver tissue. Expression of the ER stress marker glucose‐regulated protein 78 (*GRP78*) was significantly increased, and C/EBP homologous protein (*CHOP*) showed an increasing tendency in the liver tissue of the mice fed the mantle diet (Figure 2). Since recent studies have also shown that ER stress causes inflammation through the activation of nuclear factor‐kappa B (NF‐κB) (Mollica et al., 2011), we investigated the mRNA expression levels of the NF‐κB‐dependent inflammatory cytokines, interleukin‐1 alpha (*IL‐1α*) and tumor necrosis factor‐alpha (*TNF‐α*) (Figure 3). The mantle toxin increased significantly the mRNA expressions of *TNF‐α *and *IL‐1α* and showed a tendency to increase the mRNA expression of suppressor of cytokine signaling 3 (*SOCS3*), which has been reported to be correlated with the severity of inflammation (White, Cotterill, Addley, Soilleux, & Greaves, 2011). These results show that the mantle toxin causes ER stress and inflammation in the liver tissue.

### 3.3 Apoptosis in the liver tissue

**Figure 4:**
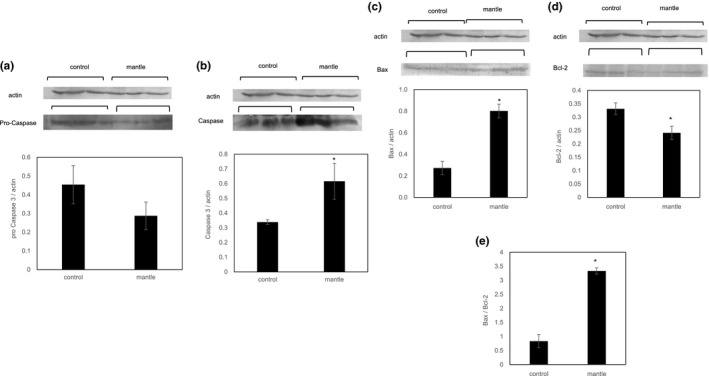
Apoptosis in the liver tissue of mice fed a diet containing mantle tissue or a control diet. After the liver was extracted with 20% sucrose solution, the amounts of procaspase 3 (a), caspase 3 (b), Bax (c), and Bcl‐2 (d) were measured by Western blot assay. The Bax/Bcl‐2 ratio (e) was calculated from (c) and (d). Bars represent the *SD*. *p < .05 relative to the control

**Figure 5:**
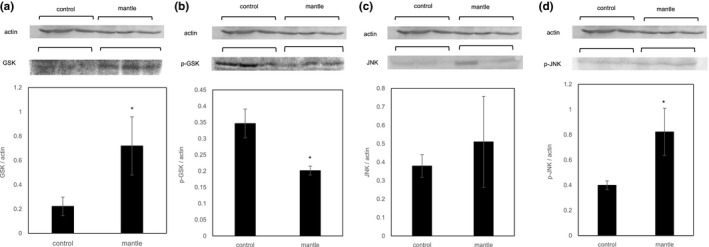
Western blot assay of the expression levels of nonphosphorylated and phosphorylated GSK‐3β (a, b) and nonphosphorylated and phosphorylated JNK (c, d). Bars represent the *SD*. *p < .05 relative to the control

Since ER stress has also been reported to cause apoptosis (Benali‐Furet et al., 2005), we investigated the protein expression levels of Bcl‐2 and Bax. Feeding of the mantle tissue increased the expression of Bax and decreased that of Bcl‐2, with concomitant upregulation of the Bax/Bcl‐2 ratio (Figure 4). In addition, the expression of caspase 3 (an activator of apoptotic DNA fragmentation) was increased, whereas that of procaspase 3 was decreased, in the liver tissue of the mice fed the mantle diet, suggesting that mantle toxin causes apoptosis in the liver tissue. To confirm this, we also investigated the GSK‐3β and JNK signaling pathways, which have been reported to be involved in ER stress‐induced apoptosis (Zhang, Yin, Song, Fan, & Hu, 2016). Mantle toxin decreased the p‐GSK‐3β level and increased the nonphosphorylated (active form) GSK‐3β level in the liver tissue (Figure 5). In addition, the mantle diet increased the phosphorylated (active form) form of JNK, suggesting that the mantle tissue activates the GSK‐3β and JNK signaling pathways. These results support the conclusion that mantle toxin causes ER stress‐induced apoptosis in liver tissue.

### 3.4 Effect of mantle tissue on gluconeogenesis

**Figure 6:**
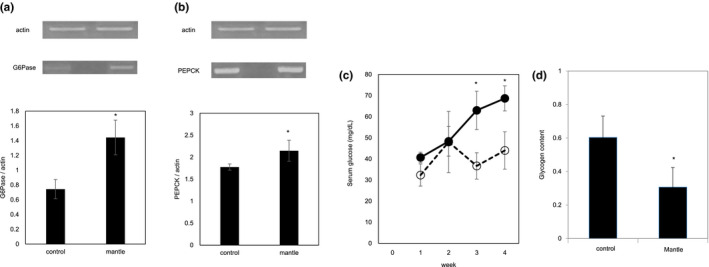
Effect of mantle tissue ingestion on the mRNA expression of G6Pase (a) and *PEPCK* (b). Liver tissue was excised from mice fed a diet containing mantle tissue and those fed a control diet, respectively, and semiquantitative RT‐PCR was performed. (c) After the feeding period, the serum glucose concentration of each mouse was measured every week. (d) Glycogen content in the liver tissue was measured. Bars represent the *SD*. *p < .05 relative to the control

GSK‐3β and JNK are involved in the insulin signaling pathway and glucose metabolism (Sharfi & Eldar‐Finkelman, 2008). An increase in the nonphosphorylated GSK‐3β level decreases the glycogen content through the inactivation of glycogen synthase, whereas p‐JNK promotes gluconeogenesis by promoting the serine phosphorylation of insulin receptor substrate in the liver tissue, resulting in the inhibition of insulin signaling. To confirm the activation of the JNK and GSK‐3β signaling pathways in the liver of mice fed mantle tissue, the glycogen content and mRNA expression levels of two gluconeogenesis enzymes (i.e., glucose‐6‐phosphatase and phosphoenolpyruvate carboxykinase) were investigated. Mantle toxin decreased the glycogen content and increased the mRNA expression of the gluconeogenesis enzymes (Figure 6), suggesting that the mantle toxin may inhibit insulin signaling in liver tissue. These results corresponded with the time‐dependent increase in serum glucose concentrations associated with mantle toxin.

## 4 DISCUSSION

In this study, mantle toxin by mice increased lipid peroxidation and decreased the SH content in the liver tissue through the lowering of the antioxidative defenses, such as the catalase, radical scavenging, and glutathione reductase activities. An increase in reactive oxygen species in the liver causes the release of calcium from the ER to the cytosol, leading to disruption of the protein folding process and the induction of ER stress. The activation of the unfolded protein response can subsequently promote inflammation and apoptosis. Thus, oxidative stress is closely associated with ER stress, inflammation, and apoptosis (Zhang, 2010). This theory corresponded with our results, where the mantle toxin caused oxidative stress, ER stress, and inflammation in the liver. However, it remains unclear whether mantle tissue directly causes oxidative stress first, because this process has also been reported to be induced by ER stress and inflammation (Passos, Ascensão, Martins, & Magalhães, 2015; Tsuneyama et al., 2002).

Previously, we had reported that the treatment of HepG2 cells with mantle tissue extract suppressed the insulin‐stimulated phosphorylation of Akt, a key protein in the insulin signaling pathway (Kariya, Takahashi, Itagaki, & Hasegawa, 2019). In addition, the mantle toxin induced ER stress in the HepG2 cells and increased the expression of p‐JNK, nonphosphorylated GSK‐3β, and SOCS3, which inhibited insulin signaling. In this study, we showed that mantle toxin caused ER stress and activated the JNK and GSK‐3β signaling pathways in liver tissue, similar to the case of HepG2 cells, suggesting that mantle toxin inhibits insulin signaling in the liver. Moreover, the toxin that causes mouse liver injury in vivo seems to be identical to the toxin that acts against the HepG2 cells. Although we are in the process of isolating the toxin from mantle tissue by using an in vivo assay system that evaluates toxicity in mice, the difficultly in preparing sufficient amounts of samples for dietary consumption and the 4–8 weeks needed for detecting the toxicity have limited our ability to obtain results quickly. Therefore, the in vitro assay system using HepG2 cells may be more useful for isolating the toxic substance and clarifying its action mechanism. Currently, we are working toward identifying this toxic substance using both in vitro and in vivo assay systems.

We had previously shown that the toxin in the mantle tissue appears to be a protein with a molecular weight of >10 kDa. Many proteinaceous toxins like abrin, ricin, and botulinum toxin have been reported (Bradberry, Dickers, Rice, Griffiths, & Vale, 2003; Cheng & Henderson, 2011; Garber, 2008; Moshiri, Hamid, & Etemad, 2016). Ricin, a water‐soluble glycoprotein, is absorbed via the blood and lymphatic vessels within 2 hr of ingestion and accumulates in the liver and spleen (Moshiri et al., 2016). The toxin in mantle tissue may likewise be absorbed and accumulated in the liver tissue, although further studies are needed to verify this.

We have shown that the intake of a diet containing mantle tissue causes the death of rats and mice. However, it remains unclear whether the indicators of liver injury, such as oxidative stress, ER stress, inflammation, and apoptosis, are essential causes leading to the death of the animals. Future studies should be undertaken to clarify whether inhibitors of oxidative stress, ER stress, inflammation, and apoptosis can suppress the mantle diet‐induced death of rats and mice.

## 5 CONCLUSIONS

In summary, we have investigated how liver tissue injuries are caused by the mantle toxin. Our results showed that the mantle toxin caused liver tissue apoptosis through the induction of oxidative stress, ER stress, and inflammation in the organ. Such injuries may be an essential cause leading to the death of the rodents, although further studies are needed to verify this.

## CONFLICT OF INTEREST

We certify that there is no conflict of interest with any financial organization.
